# Spatial heterogeneity in gender and age of fatal suicide in Iran

**DOI:** 10.34172/jrhs.2022.76

**Published:** 2021-12-28

**Authors:** Mehran Rostami, Abdollah Jalilian, Seyed Amirhosein Mahdavi, Nasser Bagheri

**Affiliations:** ^1^Deputy of Health, Kermanshah University of Medical Sciences, Kermanshah, Iran; ^2^Department of Statistics, Razi University, Kermanshah, Iran; ^3^Legal Medicine Research Center, Iranian Legal Medicine Organization, Tehran, Iran; ^4^Health Research Institute, University of Canberra, ACT 2601 Australia

**Keywords:** Completed suicide, Mapping, Spatial distribution, Iran

## Abstract

**Background:** The suicide incident has had an increasing trend in Iran over the past years. This study mainly aimed to investigate and visualize the spatial variations of registered suicide cases at the province level. A two-step modeling approach was employed in order to estimate the relative risks (RRs) and model the age of fatal suicide across provinces in Iran.

**Study design:** An applied ecological study.

**Methods:** This study used the suicide death data recorded by the Iranian forensic medicine organization from March 21, 2016, to March 20, 2018. Furthermore, a Bayesian spatial approach - Besag, York, and Mollie (BYM) model- was applied to estimate the RR of suicide across provinces in Iran.

**Results:** This risk was found to be significantly higher than the average in both men and women in the west of Iran. For women, higher population density (mean: 0.003; 95% CrI: 0.001-0.005) and lower urbanization rate of provinces (mean: -0.025; 95% CrI: -0.038, -0.012) were associated with increased RR of suicide. Based on the log-normal model fitted to the data, the overall mean age of the fatal suicide at the national level was 34 years.

**Conclusions:** The magnitude of gender and age differences was quantified, and many spatial variations were identified in suicide mortality across provinces in Iran. Given the heterogeneity in suicide mortality risk among different subgroups of age and gender, our findings point to the urgent need in developing gender- and age-specific suicide prevention strategies. Moreover, efficient allocation of healthcare resources for suicide prevention can be attained by targeting provinces with higher risk.

## Background

 Suicide is a multifactorial public health concern that involves a complex interaction among different risk factors.^1,2^ There are several theories of suicide ranging from biological and psychodynamic theories to cognitive, interpersonal, and social-ecological ones. Examples of the societal kind can be found in a study by Durkheim,^3^ with respect to the community in a study performed by Castro and Kintzle,^4^ and considering interpersonal-psychological theory in a study carried out by Joiner and Van-Orden et al,^5,6^ while Schneidman,^7^ as well as Cramer and Kapusta, focused on the individual level and socio-ecological perceptions, respectively. Durkheim defined suicide as “a term applied to all cases of death resulting directly or indirectly from a positive or negative act of the victim himself, which s/he knows will produce this result”. He also conceptualized suicide as a reaction to the intersection of social integration (the clustering of people in social and cultural groups) and regulation (the extent of rituals and customs being influenced by societal norms). Although individual-level characteristics and attitudes, as well as mental health problems, are important as primary predictors of suicide risk, societal and community factors, as well as sub-population variation, may also have a direct influence or a moderating role on suicide risk. A full review of all suicide risk theories is beyond the scope of this study; accordingly, it focused on Durkheim and socio-ecological theories to identify the risk of fatal suicide across the provinces in Iran.

 In 2016, suicide was the second most common cause of death in the 15-29-year age group globally.^1^ The global age-standardized rate of years of life lost (YLL) from suicide was estimated at 2.2% of the total YLL globally.^1^ Suicide mortality rates vary widely by country and region^1,2^ and according to the Global Burden of Disease, suicide deaths have increased by 100% in the countries of the Eastern Mediterranean Region between 1990 and 2015.^8^ In Iran, the deaths by suicide showed an increasing trend over the past three decades.^9^ A recent national study estimated the mean Iranian suicide mortality rate to be about 5 per 100 000 population in 2015.^10^ Geographical studies of suicide in the regions of Iran have been conducted; however, the spatial pattern in the whole country has not been fully investigated. Spatial analysis of suicide enables us to identify areas with greater risk of suicide and explore the potential link between area-level contextual factors and suicide risk.^11-13^ Furthermore, it visualizes the potential disparities within designated geographical areas. The Besag, York, and Mollie (BYM) model (the most commonly used Bayesian spatial model) can be used for computing suicide relative risk (RR).^11-13^ Spatial analysis of suicide mortality at regional and national levels enables policy-makers to identify geographic outcome disparity and design tailored interventions to reduce suicide risk.^11-13^ However, there is a lack of research on spatial analyses of suicide risk across the Iranian provinces.

 This study aimed at visualizing the suicide risk across all provinces over the country from 2016 to 2018 to identify geographical variations of the suicide risk pattern using a generalized linear mixed model Bayesian approach with random effects that incorporates spatial dependence among neighbouring provinces. A two-step modeling approach was employed in order to estimate the RRs and model the age of fatal suicide across provinces in Iran.

## Materials and Methods

###  Data collection and registry

 In Iran, the diagnosis and official registration of deaths are carried out by two main independent organizations of medical universities operating under the Ministry of Health and Medical Education, and the Forensic Medicine Organization (FMO) operating under the supervision of the Judiciary system of Iran.^14^ In accordance with Iranian laws, deaths by suicide, like all sudden or unexpected deaths, are reported to the Iranian FMO for forensic investigation.^14^ When a case is examined by forensic physicians and pathologists, the exact cause of death is determined and a death certificate is issued. If the test results reveal death by suicide (not related to crime), the case will be recorded in the suicide registry dataset by provincial branches of the FMO.^14,15^Therefore, it is acceptable to consider suicide cases registered by the FMO as the most valid registered data source for fatal suicide in Iran.^10,14,15^ Province-specific population data were extracted from the 2016 national census of population and housing data reported by Iran’s Statistical Centre. In this study, age, gender, and place of death of suicide cases were extracted from the registered data by the FMO suicide registry database between March 21, 2016, and March 20, 2018. This period was chosen for the study because the suicide mortality data were available to the authors only for this period at the time the study was conducted. A descriptive analysis of these data has been reported elsewhere.^15^

###  Provincial counts and ages of people who committed suicide

 A spatially structured model was first used for the provincial counts (N) to investigate the gender-specific risk of suicide among different provinces. Afterward, the study was developed to investigate the combined effect of gender and province on the age of people who committed suicide as follows:

 Let *N*_ij_ be the number of suicide cases with gender *i* = 1, 2 (where 1 and 2 stand for female and male, respectively) in province* j * = 1,... 31 and *Y*_ijk_ is the age of suicide case *k* = 1,... *N*_ij_ with gender *i* in province *j* during the study period. Moreover, let [N, Y] denote the joint distribution of $N=\left(N_{ij}:i=1,2;j=1,...31\right)$ and the individual ages of people who committed suicide

$$Y=\left(Y_{ijk}:i=1,2;j=1,...31;k=1,...N_{ij}\right)$$;

[N] and [Y|N] represent the marginal distribution of N and the conditional distribution of Y given N, respectively. Following that, [N,Y] = [N][Y|N].

 It is assumed that


$$\left[Y\left|N\right.\right]=\prod_{i=1}^{2}\prod_{j=1}^{31}\prod_{k=1}^{N_{ij}}\left[Y_{ijk}\left|N_{ij}\right.\right]$$

which is equivalent to assuming that given the number of suicide cases for each gender in each province, the age of suicide cases is independent. Therefore, to model [N, Y], it suffices to model [N] and [*Y*_ijk_*|N*_ijk_] separately.

###  A negative binomial model for the provincial counts

 It is assumed that given θ = (*θ*_ij_*:i* = 1,2; *j* = 1, …,31),


$$\left[N\left|\theta \right.\right]=\prod_{i=1}^{2}\prod_{j=1}^{31}\left[N_{ij}\left|\theta_{ij}\right.\right]$$

and [*N*_ij_|*θ*_ij_] is a negative binomial distribution with the mean $E\left(N_{ij}\right)=E_{ij}\theta_{ij}$ and variance


$$\mathrm{var}\left(N_{ij}\right)=E_{ij}\theta_{ij}\left(1+\frac{\theta_{ij}}{\alpha_{i}}\right),$$

where *E*_ij_ is the expected number of suicide cases with gender *i* in province *j* under the null model of spatial homogeneity of suicide risk, *θ*_ij_ signifies the RR of suicide for gender *i* in province *j*, and *α*_i_ > 0 denotes the dispersion parameter. If *α*_i_ → ∞, then $\mathrm{var}\left(N_{ij}\right)=E\left[N_{ij}\right]$ as for the Poisson distribution with no dispersion, while smaller values of *α*_i_ allow for larger over-dispersion.^16^ The expected number of *E*_ij_ is estimated by


$${\hat{E}}_{ij}=P_{ij}\frac{\sum_{j=1}^{31}N_{ij}}{\sum_{j=1}^{31}P_{ij}},$$

where *P*_ij_ is the population of gender *i* in province *j* with an age greater than 10 years according to the 2016 National Population and Housing Census of Iran.^17^

###  BYM model for the spatial heterogeneity of the RRs

 The RRs *θ*_ij’_s are of great interest in spatial epidemiology because they identify provinces with elevated (*θ*_ij_>1) or lower (*θ*_ij_<1) risk, compared to the whole country.^18^ In spatial epidemiology, the strength of the associations is generally estimated quantitatively by using RRs in order to identify the low- and high-risk areas. In general, if the province-specific RR is 1 (or close to 1), it indicates no association and no difference (or little difference) in suicide risk. A province-specific RR>1 indicates a higher risk in the selected province, compared to the country risk. A province-specific RR<1 indicates a lower risk in the selected province, compared to the country risk.^11,18,19^

 The log-linear model was considered to account for

$$\theta_{ij}=\exp\left(\beta_{0}+\beta_{1}^{\left(i\right)}pd_{j}+\beta_{2}^{\left(i\right)}ue_{j}+\beta_{3}^{\left(i\right)}hs_{j}+\beta_{4}^{\left(i\right)}ur_{j}+\eta_{ij}\right)$$

spatial variability. For *θ*, where *β*_0_ is the intercept, pd denotes the population density, *ue* signifies the unemployment rate, *hs* is the average household size, and *ur* represents the urbanization rate of province *j. *They were used as provincial covariates with their corresponding gender-specific regression coefficients $\beta_{1}^{\left(i\right)},...,\beta_{4}^{\left(i\right)}$ . Here, *η*_ij’_s were zero-mean Gaussian spatial random effects with assuming independence between genders implying $\mathbb{C}ov\left(\eta_{ij},\eta_{i^{\prime }j^{\prime }}\right)=0,i\neq i^{\prime }$ . A scaled version of the popular BYM model^20^ for spatial correlation among provinces results in:


$$\mathbb{C}ov\left(\eta_{ij},\eta_{i^{\prime }j^{\prime }}\right)=\left\{\begin{array}{l} \frac{1}{\tau_{BYM}}\left(1-\phi +\phi Q_{jj}^{-}\right)\;j=j^{\prime }\\ \frac{\phi }{\tau_{BYM}}Q_{jj^{\prime }}^{-}\;j\neq j^{\prime } \end{array}\right.$$

Where $Q^{-}=\left[Q_{jj^{\prime }}^{-}\right]$ is the generalized inverse of the matrix Q = [*Q*_jj’_] with *Q*_jj’ _ = 0 if provinces *j* and *j’* are not neighbours.^21^ Therefore, *τ*_BYM_ > 0 is the marginal precision parameter and 0 ≤ *ϕ* ≤ 1 signifies the proportion of the marginal variance $\frac{1}{\tau_{BYM}}$ explained by the spatial correlation among provinces.^20,21^

###  A log-normal model for the age of people who committed suicide

 To model the age of suicide cases, it is assumed that [*Y*_ijk_|*N*_ij_] is a log-normal distribution with mean $E\left(\log\left(Y_{ijk}\right)\right)=\mu +\xi_{ij}$ and variance $\frac{1}{\tau_{age}}$ ,

 where *µ* signifies the intercept, and *ξ*_ij’_s are independent and identically distributed zero-mean Gaussian random effects with variance $\frac{1}{\tau_{\xi }}$ . The term *ξ*_ij_ accounts for the effect of gender *i* and province *j* on the age of people who committed suicide and indicates gender and provinces with higher (*ξ*_ij_ > 0) or lower (*ξ*_ij_ < 0) age, compared to the whole country.

###  Fitting the model

 The Bayesian inferential framework was used to fit the considered models to the data. Accordingly, it is necessary to specify a prior distribution for any parameter in the models, reflecting an approximate and idealized concept of prior information about the parameters.^16,19^ Non-informative flat (improper uniform) priors were considered for *β*_0_; log (*α*), *µ*; log (*τ*_age_), and log(*τ*_ξ_). A Gaussian prior was also considered with a mean zero and variance 10^3^ for $\beta_{l}^{\left(i\right)},i=1,2;l=1,...,4$ , and penalized complexity priors ^22^ for log(*τ*_BYM_) and $\log\left(\frac{\phi }{1-\phi }\right)$ ^21^. The R package integrated nested Laplace approximation (INLA) was used for the computations.^23,24^ The Bayesian inference for the model was conducted using the INLA method. Markov chain Monte Carlo methods were not used, and therefore, burn-in, the number of simulation iterations, and convergence criteria for the chain were not applicable in our study.

 To assess the Bayesian goodness of fit and predictive performance of the fitted models for the provincial counts and the ages of suicide cases, the probability integral transform (PIT) values were extracted from the fitted models.^24^ If the fitted models described the data reasonably well, then the PIT values were expected to follow the standard uniform distribution (uniform distribution on [0, 1]).^19^

## Results

 The posterior mean with 95% credible interval (CrI) for the parameters of the fitted models to the provincial counts, the RRs, and the age of people who committed suicide are summarized in Table 1. It can be observed that a higher population density and a lower urbanization rate of provinces increase the RR of suicide for women. The posterior mean of *ϕ* implies that around 85% of the spatial variation in the RR of suicide is due to the spatial correlations among neighbouring provinces. Moreover, the log-normal model’s estimated parameters indicate that the country-wide average age of the fatal suicide was 34 years. The posterior mean with 95% CrI of gender-specific RRs *θ*_ij_ and spatial random effects *ξ*_ij_ for the age of people who committed suicide across Iranian provinces are reported in Table 2. In this table, an asterisk (*) indicates the gender and provinces with significantly higher or lower RRs and higher or lower age of people who committed suicide, compared to the whole country.

**Table 1:** Posterior means for the considered model parameters

	**Parameter**	**Mean**	**95% CI**
$\beta_{0}$	Intercept for the relative risks	-0.454	(-2.743, 1.833)
$\beta_{1}{}^{\left(1\right)}$	Population density for females	0.003	(0.001, 0.005)
$\beta_{1}{}^{\left(2\right)}$	Population density for males	0.001	(-0.001, 0.003)
$\beta_{2}{}^{\left(1\right)}$	Unemployment rate for females	0.048	(-0.001, 0.097)
$\beta_{2}{}^{\left(2\right)}$	Unemployment rate for males	0.025	(-0.023, 0.072)
$\beta_{3}{}^{\left(1\right)}$	Average household size for females	0.411	(-0.217, 1.038)
$\beta_{3}{}^{\left(2\right)}$	Average household size for males	0.166	(-0.461, 0.791)
$\beta_{4}{}^{\left(1\right)}$	Urbanization rate for females	-0.025	(-0.038, -0.012)
$\beta_{4}{}^{\left(2\right)}$	Urbanization rate for males	-0.007	(-0.019, 0.006)
1α	Over-dispersion of the negative binomial	4264.9	(11.9, 21667.3)
$\tau_{BYM}$	Marginal precision of the BYM model	5.977	(3.811, 8.818)
ϕ	Proportion of the structured term in the BYM model	0.855	(0.504, 0.987)
μ	Intercept for the log-normal model	3.432	(3.421, 3.443)
$\tau_{age}$	Precision for the log-normal model	5.916	(5.743, 6.092)
$\tau_{\xi }$	Precision for the spatial random effects	96.67	(60.64, 148.1)

**Table 2:** Posterior means of the gender-specific relative risk of suicide and the spatial random effect for the age of people who committed suicide across the provinces of Iran from 2016 to 2018 period

**ID**	**Province**	**Relative Risk ** ***θ***_ij_				**Spatial random effect for the age ** ***ξ***_ij_			
		**Female**		**Male**		**Female**		**Male**	
		**Mean**	**95% CI**	**Mean**	**95% CI**	**Mean**	**95% CI**	**Mean**	**95% CI**
1	Alborz	1.09	(0.89, 1.31)	1.17	(1.04, 1.32)	-0.08	(-0.16, -0.01)	0.05	(0.00, 0.10)
2	Ardebil	1.43	(1.10, 1.81)	1.39	(1.18, 1.63)	0.06	(-0.03, 0.15)	0.03	(-0.03, 0.09)
3	Bushehr	0.83	(0.58, 1.13)	0.77	(0.61, 0.94)	-0.03	(-0.16, 0.09)	-0.05	(-0.14, 0.03)
4	Chahar Mahall and Bakhtiari	1.10	(0.78, 1.49)	1.24	(1.01, 1.50)	0.12	(0.00, 0.23)	-0.01	(-0.09, 0.06)
5	East Azarbaijan	0.93	(0.78, 1.11)	1.23	(1.11, 1.35)	-0.06	(-0.13, 0.01)	0.11	(0.07, 0.15)
6	Esfahan	0.53	(0.43, 0.64)	0.82	(0.74, 0.91)	0.04	(-0.04, 0.12)	0.02	(-0.03, 0.06)
7	Fars	1.02	(0.87, 1.18)	1.26	(1.15, 1.38)	-0.11	(-0.17, -0.05)	0.03	(-0.01, 0.06)
8	Gilan	1.21	(0.99, 1.44)	1.48	(1.32, 1.65)	0.16	(0.09, 0.24)	0.23	(0.18, 0.27)
9	Golestan	1.20	(0.94, 1.49)	0.6	(0.56, 0.83)	-0.01	(-0.10, 0.07)	0.00	(-0.07, 0.08)
10	Hamadan	1.00	(0.77, 1.25)	1.79	(1.58, 2.02)	-0.05	(-0.14, 0.05)	0.07	(0.02, 0.12)
11	Hormozgan	0.63	(0.45, 0.84)	0.62	(0.50, 0.75)	-0.10	(-0.22, 0.02)	-0.12	(-0.20, -0.04)
12	Ilam	3.19	(2.45, 4.03)	2.03	(1.65, 2.46)	0.08	(-0.01, 0.17)	0.02	(-0.05, 0.10)
13	Kerman	0.81	(0.65, 0.99)	0.72	(0.62, 0.83)	-0.17	(-0.25, -0.09)	-0.07	(-0.12, -0.01)
14	Kermanshah	2.71	(2.32, 3.13)	2.13	(1.91, 2.37)	0.00	(-0.05, 0.06)	0.06	(0.02, 0.10)
15	Khuzestan	1.28	(1.10, 1.46)	1.06	(0.96, 1.17)	-0.15	(-0.20, -0.09)	-0.09	(-0.13, -0.05)
16	Kohgiluyeh and Buyer-Ahmad	2.94	(2.29, 3.67)	2.02	(1.67, 2.41)	-0.14	(-0.23, -0.06)	-0.18	(-0.25, -0.11)
17	Kordestan	1.38	(1.09, 1.71)	0.97	(0.81, 1.15)	-0.09	(-0.17, 0.00)	-0.04	(-0.11, 0.03)
18	Lorestan	2.31	(1.94, 2.71)	1.70	(1.49, 1.92)	-0.07	(-0.13, 0.00)	-0.05	(-0.1, 0.00)
19	Markazi	0.62	(0.45, 0.84)	1.01	(0.84, 1.19)	0.05	(-0.07, 0.17)	0.03	(-0.06, 0.12)
20	Mazandaran	1.02	(0.84, 1.21)	0.87	(0.76, 0.98)	0.01	(-0.07, 0.08)	0.09	(0.04, 0.14)
21	North Khorasan	1.08	(0.75, 1.47)	1.09	(0.86, 1.35)	0.08	(-0.03, 0.20)	-0.04	(-0.13, 0.04)
22	Qazvin	0.84	(0.61, 1.11)	1.15	(0.96, 1.36)	0.08	(-0.03, 0.19)	0.06	(-0.01, 0.12)
23	Qom	0.53	(0.35, 0.74)	0.84	(0.68, 1.02)	0.13	(0.01, 0.26)	0.04	(-0.03, 0.12)
24	Razavi Khorasan	0.41	(0.33, 0.50)	0.62	(0.55, 0.69)	-0.02	(-0.10, 0.06)	0.06	(0.02, 0.11)
25	Semnan	0.44	(0.28, 0.65)	0.62	(0.45, 0.81)	0.18	(0.03, 0.34)	-0.10	(-0.21, 0.01)
26	Sistan-and-Baluchistan	0.67	(0.50, 0.85)	0.42	(0.34, 0.52)	-0.13	(-0.23, -0.03)	-0.21	(-0.29, -0.13)
27	South Khorasan	0.42	(0.25, 0.65)	0.42	(0.29, 0.58)	0.14	(-0.03, 0.30)	-0.11	(-0.24, 0.01)
28	Tehran	1.01	(0.92, 1.10)	0.88	(0.83, 0.94)	0.01	(-0.03, 0.05)	0.13	(0.11, 0.16)
29	West Azarbaijan	0.76	(0.61, 0.93)	0.61	(0.52, 0.71)	-0.06	(-0.14, 0.03)	0.03	(-0.03, 0.10)
30	Yazd	0.36	(0.22, 0.53)	0.56	(0.43, 0.71)	0.06	(-0.09, 0.21)	-0.01	(-0.1, 0.09)
31	Zanjan	0.93	(0.67, 1.23)	1.38	(1.15, 1.63)	0.05	(-0.06, 0.17)	0.05	(-0.03, 0.14)

**Figure 1:**
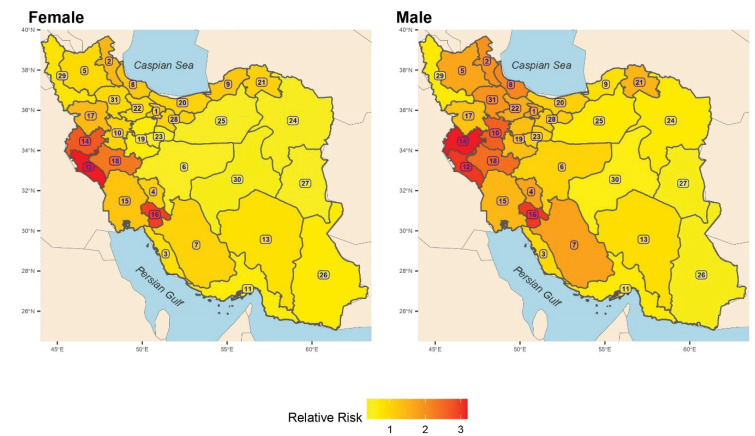
Posterior means of gender-specific relative risk of suicide across provinces in Iran from 2016 to 2018.

 The posterior mean of gender-specific provincial RRs is also mapped in Figure 1 where the spatial correlation and higher RR of fatal suicide in the western provinces are visible. Table 2 also reveals that the age of people who committed suicide was significantly lower for women, compared to the country average in Kerman (17%), Khuzestan (15%), Kohgiluyeh and Buyer-Ahmad (14%), Sistan-and-Baluchistan (13%), Fars (11%), and Hormozgan (10%) provinces. On the other hand, it was significantly higher in Semnan (18%), Gilan (16%), and Qom (13%). For men, the age of people who committed suicide in Sistan-and-Baluchistan (21%), Kohgiluyeh and Buyer-Ahmad (18%), and Hormozgan (12%) was significantly lower than that of the country average, while it was significantly higher in Gilan (23%), Tehran (13%), and East Azarbaijan (11%).

 The most noteworthy observation in Table 2 is the high suicide RR in the provinces of Ilam (more than 3 times), Kohgiluyeh and Buyer-Ahmad (around 3 times), Kermanshah (2.7 times), and Lorestan (2.3 times) for women. These values were obtained in Kermanshah (2.1 times), Kohgiluyeh and Buyer-Ahmad (2 times), and Ilam (2 times) for men.

**Figure 2:**
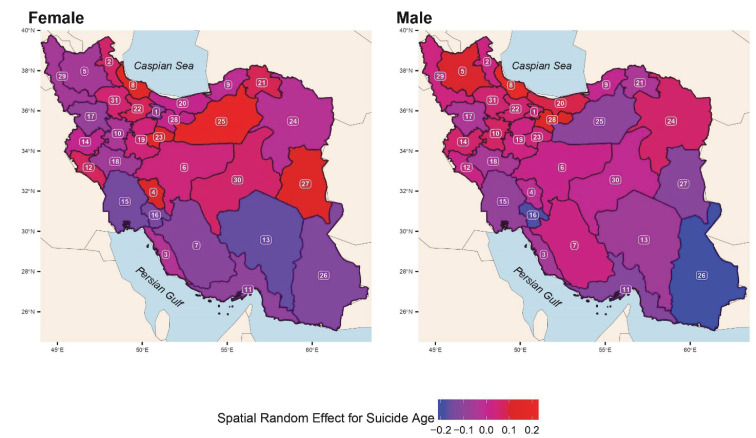
Spatial random effect for the age of people who committed suicide across provinces in Iran from 2016 to 2018.

**Figure 3:**
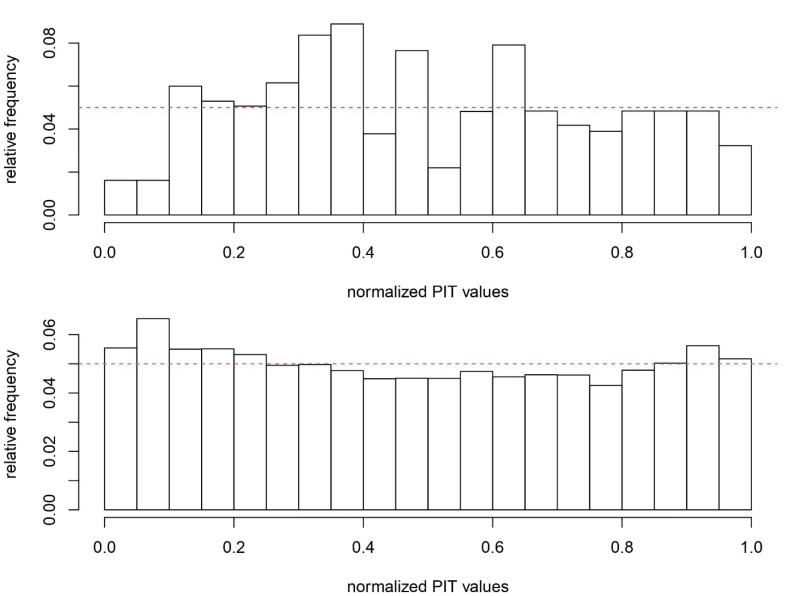
Histograms of the probability integral transformation values extracted from the fitted model to the provincial counts (top) and the age of people who committed suicide (bottom).
 Dashed line = the density function of the standard uniform distribution.

Figure 2 shows the posterior mean of the spatial random effect in the log-normal model for suicide, while the histograms of the fitted models to the provincial counts (*N*_ij’_s) and ages of the suicide cases (*Y*_ijk’_s) can be observed in Figure 3. The PIT values for the fitted log-normal model to the age of people who committed suicide are close enough to the realizations from the standard uniform distribution. However, the PIT values for the fitted model to the provincial counts show some discrepancies with what is expected from realizations from the standard uniform distribution. This indicates that there is room for improving the model fit to the provincial counts by including more relevant covariates in the model for the RRs or considering a model with more complex spatial correlation than the BYM model.

## Discussion

 The findings demonstrate the spatial correlation and higher RR of fatal suicide in western provinces in Iran. The risk of suicide mortality among females was the lowest in Yazd, South Khorasan, and Razavi Khorasan provinces. In addition, the risk of suicide mortality among males was the lowest in South Khorasan, as well as Sistan-and-Baluchistan provinces. In both genders, the age of people who committed suicide was significantly lower among the residents of Kerman, Khuzestan, Kohgiluyeh and Buyer-Ahmad, as well as Sistan-and-Baluchistan provinces, compared to the national level. By contrast, in both genders, the age of people who committed suicide was significantly higher among the residents of Gilan province, compared to the overall mean age of people who committed suicide at the national level. Moreover, the age of people who committed suicide was significantly higher among the females in Semnan and Qom provinces, compared to that at the national level.

 In Iran, the overall age-standardized rate of suicide death has decreased from 4.32 per 100 000 to 2.78 per 100 000 population between 1990 and 2015.^25^ However, this decreasing trend was not consistent during this period. Suicide mortality has slightly increased in Iran during 2006-2010, according to the national suicide registry data.^25,26^ Many studies were previously conducted to quantify the risk of provincial suicide disparities and visualize the spatial pattern of suicide at the province level.^11-13^ Health policy-makers are often interested in visualizing the spatial distribution of health outcomes as it can help them to determine unmet needs areas and better resource allocation.^11-13^

 As far as the authors are aware, this study provided the first comprehensive Bayesian evidence on the spatial heterogeneity of suicide mortality across the Iranian provinces. Our findings are in line with the results of previous studies that reported the most suicide death cases in the western provinces of Iran.^26,27^ A possible explanation might be related to the combined impacts of rapid urbanization, low socio-economic status, and cultural diversity,^25,26,28,29^ which intensified conflicts between traditions and modernity in this part of the country. As shown by the study, the lowest risks of suicide mortality among females were related to South Khorasan, Yazd, and Razavi Khorasan provinces. A possible explanation might be that the Human Development Index for these provinces was higher, compared to that in the rest of Iranian provinces in 2016.^17^ Moreover, the lowest risks of suicide mortality among males were related to South Khorasan, as well as Sistan-and-Baluchistan provinces which are neighbouring provinces located in the south-eastern region of Iran. Interestingly, these two provinces are less developed and have a strongly patriarchal culture, which might contribute to the lower risk of suicide of men in this region. The spatial analyses of suicide risk by provinces generate new evidence for policy planning and suicide prevention activities.

 Globally, suicide mortality rates for men were higher than women across all regions, countries, and age groups, except for the 15-19 age groups.^1^ At the national level, the annual suicide mortality rate was higher among individuals aged 15-24 years between 1990 and 2015.^25^ The results presented in Table 2 show that the age of suicide varies in different regions of Iran. In both genders, the age of people who committed suicide was significantly lower among the residents of Kohgiluyeh and Buyer-Ahmad, Sistan-and-Baluchistan, Kerman, Khuzestan, and Hormozgan provinces than the overall mean age of people who committed suicide at the national level. However, these findings should be interpreted with some caution. It seems that the South of the country can be considered a new source of YLL attributable to suicide. By contrast, the age of people who committed suicide among the residents of Gilan province and females in Semnan province was significantly higher than the overall mean age of people who committed suicide at the national level. An earlier and recent systematic review from January 2008 to January 2018 showed that the mean age of suicide in Iran was 29.8 years (age range: 27.7-31.8 years) for men and 27.4 years (age range: 25.8-28.9 years) for women.^30^ These findings demonstrate that suicide among Iranian women occurred at younger ages than men over the past decade.^15,30^ However, identifying the exact causes of this heterogeneity is beyond this paper’s scope, and further research is warranted. On the other hand, the age range reported was significantly lower than that in our findings (34 years). Three reasons explain this difference. One is that our study included only fatal (completed) cases of suicide; secondly, the data were analyzed from 2016 to 2018, while the above review^30^ included both fatal and attempted suicide cases and used data over 10 years (2008 to 2018). A final point is that this review included mostly province-level studies, while the present study was conducted on a national level.

 The 2015 national mental health survey of the 36 000 adult population estimated the prevalence of mental disorders based on the Diagnostic and Statistical Manual of Mental Disorders, 5^th^ Edition criteria and found it to be 23.44%.^31^ The survey’s results demonstrated an increasing prevalence of national mental disorders, compared to the 1999 study.^31^ Previous findings would suggest that policy-makers consider the implementations to decrease the incidence of suicide in Iran that include improved social equity, mitigated economic and unemployment problems, promotion of healthy lifestyles, provision of community mental health services, as well as designing a surveillance system for early detection or screening for severe depression, enhanced coping skills for families and at-risk people that would also promote position and participation of the female population in the community, and job market supporting a smooth transition from traditional society to modernity.^32^ Given the heterogeneity in suicide mortality risk among different subgroups of age and gender, our findings point to the urgent need in developing gender- and age-specific suicide prevention strategies. Moreover, efficient allocation of healthcare resources for suicide prevention can be attained by targeting provinces with higher risk.

 Our study had several strengths, including the use of valid registered suicide data, Bayesian statistics to estimate geographical distribution of suicide, large sample size, and geospatial modeling of suicide risk at the national scale. Compared to the overall estimation, using the BYM model for mapping men’s and women’s suicide patterns produces more accurate estimates, even if the age-standardized mortality rates do not account for possible spatial dependence among the neighbouring provinces and only provides raw estimates of age-standardized suicide rates across provinces in Iran.

 Our study also had some limitations. First, deaths in rural and remote areas are not always referred to FMO for further investigations. Second, given the nature of the data available to us, no information was available on mental disorders, marital status, occupation, or educational levels, all of which could contribute to suicide deaths. Third, given the time and resources required to complete autopsies, there is a time lag between the actual death date and when the cause of death is updated in FMO records.^15^ Fourth, it is likely that suicide death data could be under-registered due to Iranian society’s conservative context.^33^ Finally, there was no access to county-level data, which can be considered another limitation. In this regard, it is recmmended that future researchers conduct county-level spatial analysis.

## Conclusions

 The findings of this study unraveled the evidence of gender and age differences, as well as spatial variations in suicide mortalities across provinces in Iran. This information would inform the staff at the Ministry of Health and other provincial-level administrative centres dealing with health-related matters targeting interventions to the communities at highest risk and suggests that the implementation of a suicide risk prediction model into a policy setting may aid in early prevention and detection of people at high risk. In general, keeping up-to-date national policies, strategies, and programs relating to Iran’s suicide prevention activities are necessary for the future.

## Conflict of Interests

 The authors declare that they have no conflict of interest.

## Ethical Considerations

 The study protocol was reviewed and approved by the Ethics committee of Kermanshah University of Medical Sciences, Kermanshah, Iran (IR.KUMS.REC.1398.330). Given the anonymized and de-identified nature of the suicide-related mortality data, no informed consent was required for this study.

## Funding

 This study received no specific grant from any funding agency in public, commercial, or not-for-profit sectors.

**Highlights**

- The suicide relative risk (RR) was higher in both men and women in the west of Iran.
- A higher population density and a lower urbanization rate across the provinces in Iran increase the RR of suicide among women.
- Based on the log-normal model, the overall mean age of fatal suicide at the national level was 34 years.

## References

[1] Naghavi M (2019). Global, regional, and national burden of suicide mortality 1990 to 2016: systematic analysis for the Global Burden of Disease Study 2016. BMJ.

[2] Murray CJ, Barber RM, Foreman KJ, Abbasoglu Ozgoren A, Abd-Allah F, Abera SF (2015). Global, regional, and national disability-adjusted life years (DALYs) for 306 diseases and injuries and healthy life expectancy (HALE) for 188 countries, 1990-2013: quantifying the epidemiological transition. Lancet.

[3] Durkheim É. Le Suicide. Paris: F. Alcan; 1897.

[4] Castro CA, Kintzle S (2014). Suicides in the military: the post-modern combat veteran and the Hemingway effect. Curr Psychiatry Rep.

[5] Joiner TE. Why People Die by Suicide. 1st ed. Cambridge, MA: Harvard University Press; 2007.

[6] Van Orden KA, Witte TK, Cukrowicz KC, Braithwaite SR, Selby EA, Joiner TE Jr (2010). The interpersonal theory of suicide. Psychol Rev.

[7] Schneidman ES (1981). A psychological theory of suicide. Suicide Life Threat Behav.

[8] Rezaeian M, Moosa Khan M (2020). Suicide prevention in the Eastern Mediterranean region. Crisis.

[9] Naghavi M, Shahraz S, Sepanlou SG, Dicker D, Naghavi P, Pourmalek F (2014). Health transition in Iran toward chronic diseases based on results of Global Burden of Disease 2010. Arch Iran Med.

[10] Izadi N, Mirtorabi SD, Najafi F, Nazparvar B, Nazari Kangavari H, Hashemi Nazari SS (2018). Trend of years of life lost due to suicide in Iran (2006-2015). Int J Public Health.

[11] Mahaki B, Mehrabi Y, Kavousi A, Mohammadian Y, Kargar M (2015). Applying and comparing empirical and full Bayesian models in study of evaluating relative risk of suicide among counties of Ilam province. J Educ Health Promot.

[12] Rostami M, Jalilian A, Ghasemi S, Kamali A (2016). Suicide mortality risk in Kermanshah province, Iran: a county-level spatial analysis. Epidemiol Biostat Public Health.

[13] Rostami M, Jalilian A, Rezaeian S, Kamali A (2019). Gender and spatial disparities of suicide mortality risk in Kermanshah province, Iran: a brief report Dr. Sulaiman Al Habib Medical Journal.

[14] Rostami M, Nazparvar B, Rezaeian S (2018). Differences among official statistics of mortality rates in Iran. J Occup Health Epidemiol.

[15] Mahdavi SA, Rezaeian S, Rostami M (2020). Profile of fatal suicide in Iran: a report from the Iranian forensic medicine between 2016 and 2018. Acta Med Iran.

[16] Lawson AB. Bayesian Disease Mapping: Hierarchical Modeling in Spatial Epidemiology. 3rd ed. Chapman and Hall/CRC Press; 2018.

[17] Statistical Center of Iran. Population and Housing Censuses. Tehran: Statistical Center of Iran Publications; 2016. Available from: https://www.amar.org.ir/english/Population-and-Housing-Censuses.

[18] Martínez-Beneito MA, Botella-Rocamora P. Disease Mapping: From Foundations to Multidimensional Modeling. Hoboken, NJ: CRC Press; 2019.

[19] Blangiardo M, Cameletti M. Spatial and Spatio-Temporal Bayesian Models with R-INLA. Hoboken, NJ: John Wiley & Sons; 2015.

[20] Besag J, York J, Mollié A (1991). Bayesian image restoration, with two applications in spatial statistics. Ann Inst Stat Math.

[21] Riebler A, Sørbye SH, Simpson D, Rue H (2016). An intuitive Bayesian spatial model for disease mapping that accounts for scaling. Stat Methods Med Res.

[22] Simpson D, Rue H, Riebler A, Martins TG, Sørbye SH (2017). Penalising model component complexity: a principled, practical approach to constructing priors. Stat Sci.

[23] Rue H, Riebler A, Sørbye SH, Illian JB, Simpson DP, Lindgren FK (2017). Bayesian computing with INLA: a review. Annu Rev Stat Appl.

[24] Gómez-Rubio V. Bayesian inference with INLA. United States: CRC Press; 2020.

[25] Ghodsi Z, Moghaddam SS, Vezvaei P, Yoosefi M, Rezaei N, Saadat S (2020). The mortality rate from self-harm in Iran. Public Health.

[26] Kiadaliri AA, Saadat S, Shahnavazi H, Haghparast-Bidgoli H (2014). Overall, gender and social inequalities in suicide mortality in Iran, 2006-2010: a time trend province-level study. BMJ Open.

[27] Nazari Kangavari H, Shojaei A, Hashemi Nazari SS (2017). Suicide mortality trends in four provinces of Iran with the highest mortality, from 2006-2016. J Res Health Sci.

[28] Rezaeian M (2010). Suicide among young Middle Eastern Muslim females. Crisis.

[29] Rostami M, Jalilian A, Rezaei-Zangeneh R, Salari A (2016). Factors associated with the choice of suicide method in Kermanshah Province, Iran. Ann Saudi Med.

[30] Sharif Nia H, Heidari M, Naghavi N, Lehto RH, Haghdoost AA, Jafari-Koulaee A, et al. Age changes and suicidal activity in Iran over the past decade: a systematic review and meta-analysis. Omega (Westport). 2020:30222820966934. 10.1177/0030222820966934 33106088

[31] Noorbala AA, Faghihzadeh S, Kamali K, Bagheri Yazdi SA, Hajebi A, Mousavi MT (2017). Mental health survey of the Iranian adult population in 2015. Arch Iran Med.

[32] Rostami M, Jalilian A, Rezaei-Zangeneh R, Jamshidi T, Rezaeian M (2016). Suicide pattern in Kermanshah province, west of Iran: March 2012–March 2013. Middle East J Fam Med.

[33] Karamouzian M, Rostami M (2019). Suicide statistics in Iran: let’s get specific. Am J Mens Health.

